# Adaptive connectivity control in networked multi-agent systems: A distributed approach

**DOI:** 10.1371/journal.pone.0314642

**Published:** 2024-12-02

**Authors:** Marko Križmančić, Stjepan Bogdan

**Affiliations:** University of Zagreb Faculty of Electrical Engineering and Computing, Zagreb, Croatia; University of Shanghai for Science and Technology, CHINA

## Abstract

Effective communication is crucial for the performance and collaboration within cooperative networked multi-agent systems. However, existing literature lacks comprehensive solutions for dynamically monitoring and adjusting communication topologies to balance connectivity and energy efficiency. This study addresses this gap by proposing a distributed approach for estimating and controlling system connectivity over time. We introduce a modified consensus protocol where agents exchange local assessments of communication link quality, enabling the estimation of a global weighted adjacency matrix without requiring centralized information. The system’s connectivity is measured using the second smallest eigenvalue of the communication graph Laplacian, commonly referred to as algebraic connectivity. Additionally, we enhance the consensus protocol with an adaptive mechanism to expedite convergence, irrespective of system size or structure. Furthermore, we present an analytical method for connectivity control based on the Fiedler vector approximation, facilitating the addition or removal of communication links. This method adjusts control parameters to accommodate minor variations in link quality while reconfiguring the network in response to significant changes. Notably, it identifies and eliminates energy-consuming yet non-contributory links, improving long-term connectivity efficiency. Simulation experiments across diverse scenarios and the number of agents validate the efficacy of our proposed algebraic connectivity estimation and tracking strategy. Results demonstrate robust connectivity maintenance against external disturbances and agent failures, underscoring the practical utility of our approach for real-world multi-agent systems.

## Introduction

With the rapid advances in computer hardware miniaturization and the continuous development of communication infrastructure, distributed networked systems have emerged as an increasingly popular solution to the various challenges of modern life. In such systems, each entity represents an autonomous intelligent agent that contributes its part to the collective objective. Therefore, they are often referred to as multi-agent systems (MAS) [1]. These systems have a wide range of applications in various domains, as distributing the workload among agents greatly simplifies complex tasks such as exploration [2], transportation [3], construction [4, 5], search and rescue [6], and even interacting with animal societies [7, 8].

Furthermore, MAS are frequently employed to model intelligent wireless sensor networks. The EU-funded project WatchPlant [9, 10] that inspired the work herein leverages this approach to develop a biohybrid sensor network for environmental monitoring. This project integrates AI-equipped wireless devices with living plants, essentially transforming them into “smart bio-sensors.” By combining interdisciplinary expertise in computer science, biology, electronics, and environmental science, WatchPlant aims to create a network of intelligent plant-based sensors capable of distributed data processing, decision-making, and environmental modeling, thereby enabling plants to contribute to a broader multi-agent system for urban sustainability.

Generally, the primary objective of distributed algorithms is to allow agents to achieve a common goal despite their diverse capabilities and limited knowledge of the environment. In light of these limitations, effective communication and coordination play a vital role in determining the system’s success. Several studies have shown that information exchange between agents directly impacts the system’s performance, including convergence speed and collaboration effectiveness [11, 12]. However, expansive communication requires increasing the number of connections between agents, which increases energy consumption and can lead to degraded control due to collisions and bandwidth constraints. To deal with conflicting demands of broad information exchange and minimizing negative effects, methods for controlling the system’s connectivity and dynamically adapting it to the current situation of the environment and desired performance are necessary.

**Fig 1:**
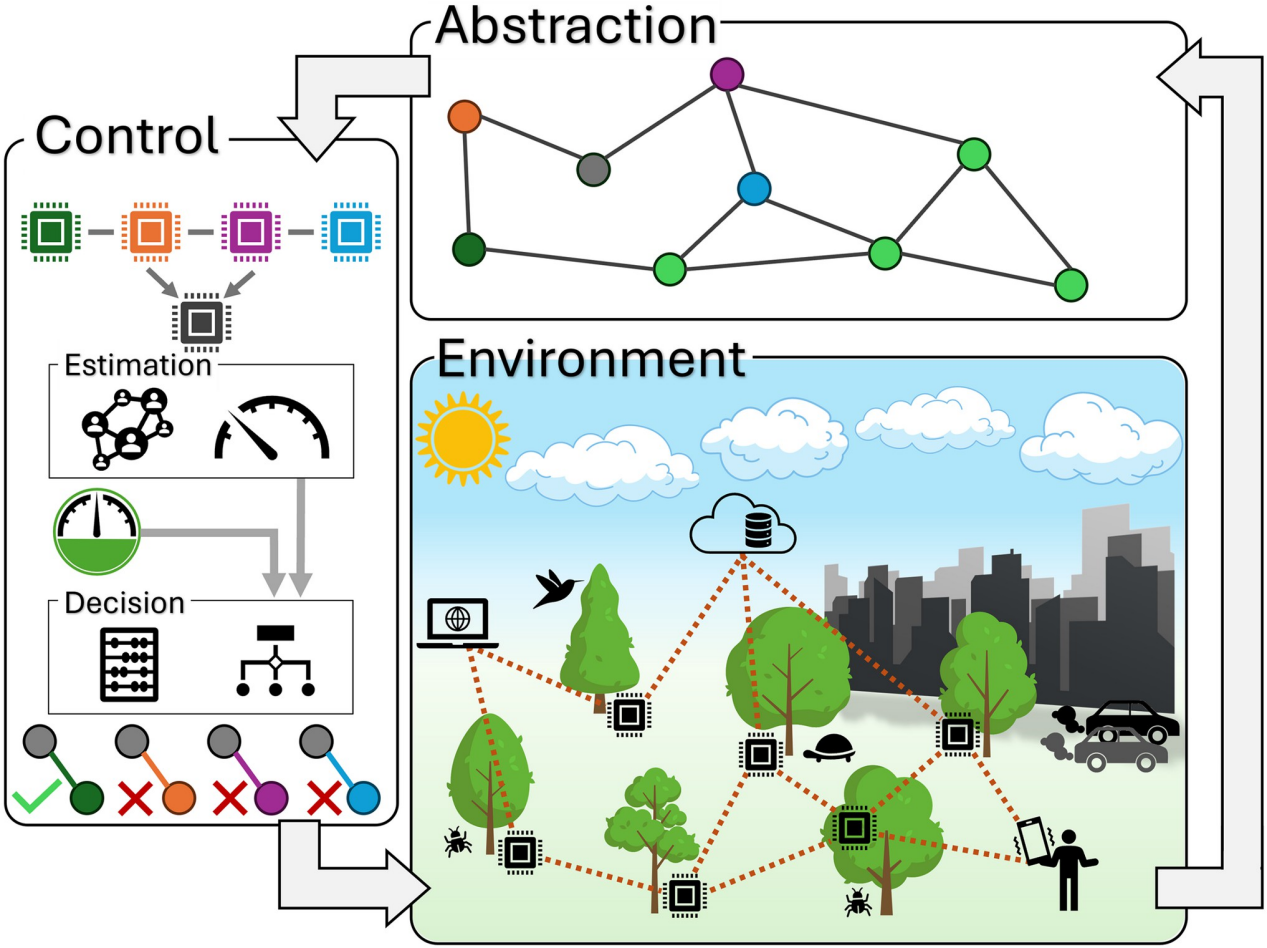
Connectivity control schematic. A wireless network of sensors, computers, and user devices monitors the environment. The sensor network is abstracted as a communication graph. Using the information from neighbors, each node estimates the network state and decides which links to modify to maintain the desired connectivity.

The effective control of communication potential in a multi-agent system can be precisely described using graph theory. By representing the communication network between agents as a graph, where each edge denotes a communication channel between two agents, we can use graph properties to capture the relevant states of all agents and their communication channels. This provides valuable insights for the topology management algorithm that monitors the state of the network and adjusts connectivity by adding or removing links. Our approach to the described objective is summarized in Fig 1. The environment of networked bio-sensors is abstracted as an undirected communication graph. Each node (sensor) receives information from its neighbors and estimates the network state which allows for a collective decision on which links to modify to reach and maintain the desired connectivity.

Along with energy consumption and topology management issues, other relevant challenges in multi-agent systems include scalability and fault tolerance [13]. In real-world scenarios, communication can degrade rapidly due to channel quality changes, obstacles, agent failures, and attacks. Distributed algorithms must be designed to be resilient to such unexpected changes and to allow the system to rapidly reconfigure to maintain desired performance and uninterrupted execution of the given task.

### Related work

Consensus algorithms are widely used in distributed algorithms for coordination and cooperation in multi-agent systems [14]. They are often employed in auction protocols, clock synchronization, sensor fusion, etc. In the context of topology management, consensus is mainly used for connectivity maintenance, i.e. preserving the network’s initial structure or ensuring that the communication graph remains connected even when environmental perturbations occur. For example, consider a robotic team exploring an unknown area. During exploration, their formation must adapt to fit corners and tight spaces while remaining connected. In such cases, connectivity control laws are typically incorporated within the motion controllers [15–19]. A similar approach is used in sensor networks, where stationary nodes maintain connectivity by adjusting the communication radius in case of failing sensors [20]. However, these methods use only Euclidean distances between agents to model network state and ignore other unpredictable phenomena that affect wireless network performance. In contrast, our method does not rely on distance criteria but on a generalized channel quality measure.

Another important aspect of topology management is connectivity determination. Distributed methods are used to gain insight into the overall topology of the multi-agent system, using only limited local knowledge of individual agents. In our work, we use a variation of a trust-inspired consensus algorithm [21] to estimate the network structure through information exchange between agents. Most distributed methods for determining connectivity rely on power iteration [22–24], where algebraic connectivity is computed from iteratively updated normalized eigenvectors corresponding to the Laplacian matrix of the graph. Wheeler *et al*. [25] combined a switching signal that determines the network topology with the consensus algorithm to rule out fake nodes and achieve agreement. Zhang *et al*. [26] extended a well-known centralized method by estimating two unavailable terms using high-pass consensus filters. They theoretically proved the globally asymptotic convergence, but the technique applies only to static graphs. An approach for dealing with time-varying directed graphs in underwater sensor networks was presented in [27]. The authors proposed a Q-learning method with an adaptive learning rate to estimate the probabilistic adjacency matrix and the corresponding generalized algebraic connectivity.

Determining the connectivity of a graph can be achieved through various metrics. Algebraic connectivity is often utilized in networked system research as it is a fundamental gauge of system performance [28] and has a direct impact on the speed of convergences in the consensus protocol [29, 30]. Furthermore, a higher value of algebraic connectivity improves the graph’s robustness to node and link failures [31]. Algebraic connectivity can be trivially increased by adding more links in the graph, but determining which links lead to the quickest increase in connectivity is a challenging problem. To address this problem, Ghosh and Boyd [32] proposed a simple greedy heuristic based on the first-order approximation of the derivative of algebraic connectivity. Their approach demonstrated that the connectivity can be maximized by adding a link between two nodes with the largest difference between their corresponding elements of the Laplacian eigenvector. The second-order approximation was later presented in [33]. Kim and Mesbahi [34] presented a computationally efficient bisection algorithm that yields better results than the simple greedy approach in a similar running time. Another heuristic involves connecting the two nodes with the minimum degrees and maximum distance from each other [35]. Authors in [36] provided necessary and sufficient conditions to determine redundant links in undirected graphs. In certain cases, removing these links improves the convergence rate of the consensus algorithm. Finally, a convolutional neural network was used in [37] to find the optimal placement of relay agents that maximizes the connectivity.

In real networks, increasing the number of connections increases the frequency of communication medium usage, leading to a potentially higher number of transmission collisions, transmission delays, degradation of throughput, and excessive energy consumption [38]. Balancing the need for maximum connectivity and robustness with minimum network cost is a challenging convex optimization problem [39], and selecting the right set of links to modify to achieve a desired connectivity level is NP-hard [40].

In [41], the authors investigated this problem in the context of optimizing cooperative multi-vehicle localization. They formulated the constrained maximization problem as a Mixed Integer Semidefinite Program (MISDP). Using the novel maximum cost heuristic, their method can quickly find good solutions with tight upper bounds. Alenazi *et al*. [42] focused on topology optimization in the context of telecommunication operators, where the cost of adding a link is mainly associated with its length. They developed an iterative algorithm that selects the desired number of links to add while balancing between improving network connectivity and minimizing the total cost. A trade-off between area coverage and connectivity was considered in [43]. The problem is divided into parts, each with a cost function that is jointly optimized using a gradient descent controller. Algebraic connectivity and its partial derivatives were calculated distributively. Some works also explore minimizing connectivity while keeping the graph connected, for instance, to slow the spread of disease [44] or cascading power failure [45].

This study does not aim to solve the problem of optimal network design, since the definitions of cost and optimal connectivity depend on the particular use case. Instead, we focus on addressing the challenge of distributively estimating network topology and controlling it in time to track the desired connectivity while rejecting disturbances. This challenge is not very well addressed in the literature. One of the few examples compares methods from previous studies [32, 34] and proposes a hybrid algorithm based on the bagging random forest classifier to select the best method depending on the current topology [46]. While this approach successfully exploits the advantages of both methods, it does not account for node failures and struggles to provide reliable results in a real distributed system. Dutta *et al*. [47] investigate connectivity tracking as part of controller design for unmanned aerial vehicle formation. They propose an offline method for computing the controller gains necessary to maintain a set connectivity level. This offline method is then repeatedly re-run to update the gains for the changing reference connectivity. In another study, the authors present a distributed stochastic power iteration method for estimating algebraic connectivity in realistic scenarios [48]. The estimated connectivity is used in a simple connectivity controller that adjusts the transmission power of the nodes. However, it assumes equal power transmission for all nodes, limiting its efficiency. A recent study focuses on controlling algebraic connectivity by adding and removing links until an approximate *k*-regular graph is constructed [49]. Two distributed algorithms are proposed: one relies on agreement between agents, while the other lets nodes modify their edges independently. The second algorithm is faster than state-of-the-art methods, and both offer resilience to node attacks by preserving connectivity. However, their applicability is limited to the algebraic connectivity values and structure of k-regular graphs.

Our previous research also tackled this problem by distributively estimating MAS topology through consensus and modifying links using a probabilistic approach [50].

### Paper contributions

In this work, we propose a novel network connectivity control method for multi-agent systems that enables them to accurately track the reference value of connectivity over time. By assuming an external reference generator, our method allows the system to balance the need for better performance or lower energy consumption depending on the user’s requirements. We measure connectivity using the second smallest eigenvalue of the communication graph’s Laplacian matrix, also known as algebraic connectivity. Connectivity is estimated in a decentralized manner using a trust-inspired consensus algorithm applied to the adjacency matrix. Our method relies solely on local information about the immediate neighbors of the nodes and does not require global information about the initial communication graph. We also consider the actual time-varying quality of the communication channels between agents, which can be modeled with a generalized function. Building on our prior work [51], we enhance connectivity estimation with a novel adaptation mechanism and a convergence detection method to improve convergence speed.

To achieve the desired connectivity value, our proposed connectivity feedback controller analytically selects which links in the network to add or remove based on the Fiedler vector approximation [32]. This approach is additionally adapted for reconfiguring the communication graph by removing connections that consume energy but do not contribute to the overall connectivity. Unlike existing work, our method is robust to interference and agent failures. As we show in the results section, a multi-agent system can detect these adverse events, exclude failing agents from communication, and quickly reconfigure itself to maintain the desired connectivity. The proposed method is successfully validated in simulations with different scenarios and system sizes. In summary, the main contributions of this paper are:

1. An adaptive consensus-based algorithm for distributed estimation of the communication network in multi-agent systems.
2. An analytical connectivity feedback controller based on Fiedler vector approximation for managing active and redundant links.
3. A distributed topology control method resilient to disturbances and agent failures for dynamically tracking the desired algebraic connectivity.

### Paper structure

The remainder of the paper is structured as follows. First, we give basic notations and background on the graph theory required to formulate our approach. The following section introduces a definition of a multi-agent system, necessary assumptions, and a detailed description of the distributed connectivity control problem. Then, we present our solution for estimating the graph topology and controlling the connectivity by modifying appropriate links, followed by a description of the experiments and results of the proposed approach. We conclude with a brief summary and some comments on possible future research directions.

## Preliminaries on graph theory

Herein, we introduce essential graph theory notations and concepts used in this paper. A graph G=(V,E) consists of a set of vertices (or nodes) V={1,2,…,n}, where *n* is the total number of vertices in the graph, and a set of edges E⊂V×V that represent connections between them. The vertices connected to the vertex *i* are called neighbors of vertex *i* and we denote them by a set $N_{i}=\left\{v_{j}\in V:e_{ij}\in E\right\}$. If the existence of the edge *e*_*ij*_ implies the existence of *e*_*ji*_, the graph is undirected. Otherwise, each edge has an assigned direction and the graph is directed. A graph G is connected if there exists a path over one or more edges between any two of its vertices.

The adjacency matrix $\text{A}\in R^{n\times n}$ defines the connections of the vertices in the graph. If vertex *i* is adjacent to vertex *j*, then adjacency matrix element *a*_*ij*_ is equal to 1, otherwise *a*_*ij*_ = 0. In weighted graphs, adjacency elements can assume a range of real values *a*_*ij*_ ∈ [0, 1], if $e_{ij}\in E$. The adjacency matrix is symmetric *a*_*ij*_ = *a*_*ji*_ for an undirected graph. The degree matrix **D** = diag(*d*_1_, …, *d*_*n*_) is a diagonal matrix of vertex degrees *d*_*i*_ that are calculated as $\;d_{i}=\sum_{j=1}^{n}a_{ij}$.

The Laplacian matrix is defined as

$$\begin{array}{c} \text{L}=\text{D}-\text{A}, \end{array}$$ (1)

with *l*_*ii*_ = *d*_*i*_ and *l*_*ij*_ = *l*_*ji*_ = −*a*_*ij*_. The Laplacian matrix is particularly interesting in research on graph connectivity. For an undirected weighted graph G with nonnegative weights,

1. all eigenvalues of **L** have real nonnegative values,
2. **L** is a symmetric positive semidefinite matrix,
3. for the column vector **1**, where all elements are equal to 1, and the zero vector **0**, it holds that **L1** = **0**.

Consequently, one eigenvalue of the Laplacian matrix is zero and can be denoted by λ_1_ = 0 and the others can be arranged in a nondecreasing order:

$$\begin{array}{c} \lambda_{1}\leq \lambda_{2}\leq \ldots \leq \lambda_{n}. \end{array}$$ (2)

The second smallest eigenvalue of the Laplacian matrix, λ_2_, has received much attention in the literature. For example, it is related to the number of connected components of the graph, its sparsity cuts, and linear embeddings [32]. Fiedler [52] first showed that the undirected graph is connected if and only if $\lambda_{2}>0$, which is why this value is often referred to as the algebraic connectivity of the graph, or the Fiedler value.

**Fig 2:**
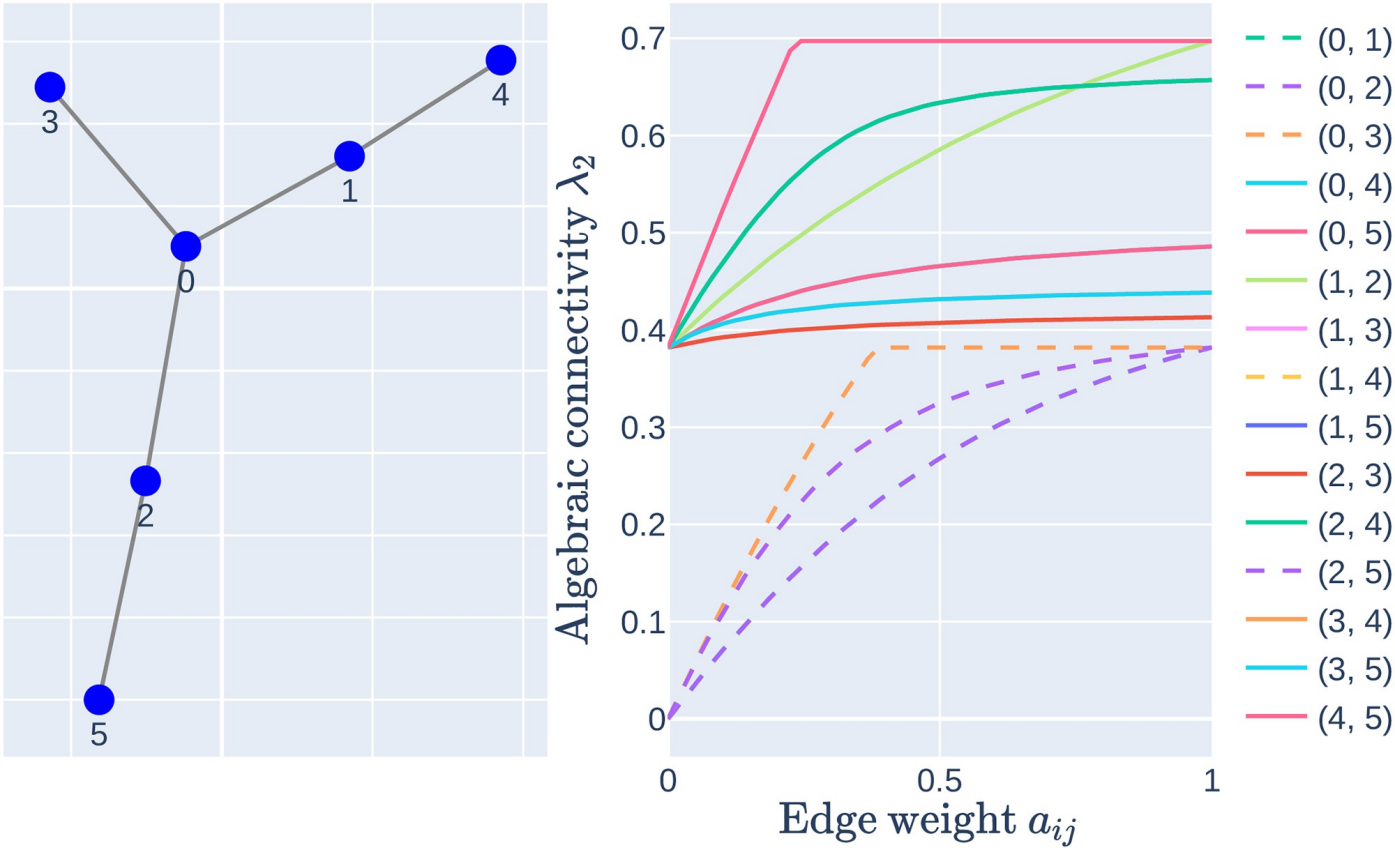
Relationship between algebraic connectivity and edge weights. *Left*: Example graph with 6 vertices. *Right*: Values of algebraic connectivity as a function of the edge weight between vertices *i* and *j*. Solid lines denote that the respective edge is being added (weight changes from 0 to 1), while dashed lines represent gradual edge removal.

The relationship between the graph’s properties and algebraic connectivity is difficult to express analytically. In the case λ_2_ is simple, i.e. there are no multiple Laplacian eigenvalues of the same value, Kirkland and Neumann [53] have shown that algebraic connectivity is a non-decreasing and concave down function of the weight of a single edge, but offer no analytical solution in the general case. Fig 2 demonstrates the complex relationship between the algebraic connectivity and weights of the edges.

For some specific graph families, such as star, cycle, or complete graphs, the algebraic connectivity can be determined by exact expressions as shown in Table 1. The absolute upper bound of the algebraic connectivity is equal to the number of vertices in the graph and is achieved with a complete graph $K_{n}$ where every vertex is connected to every other vertex, $\lambda_{2}\left(K_{n}\right)=n$. In the general case, the bounds of algebraic connectivity λ_2_ depend not only on the number of vertices but also on other parameters of the graph, including the number of edges and minimal degree of the graph, as shown in Table 2. A comprehensive overview of the graph’s spectral properties can be found in [54], while [55] offers an extended list of algebraic connectivity bounds and their relation to other properties.

**Table 1:** Algebraic connectivity *a*(*G*) for various graphs.

Graph G	Algebraic connectivity, λ_2_
Complete graph $K_{n}$	*n*
Path $P_{n}$	2(1 − cos(*π*/*n*))
Cycle $C_{n}$	2(1 − cos(2*π*/*n*))
Bipartite complete graph $K_{p,q}$	min(*p*, *q*)
Star $K_{1,q}$, q>1	1

**Table 2:** Bounds to algebraic connectivity related to basic graph’s parameters.

Graph’s parameter	Algebraic connectivity λ_2_
Number of vertices *n* *	λ_2_ ≤ *n* − 2
Number of edges *m* *	$\lambda_{2}\leq (-1+\sqrt{1+2m})$
Minimal degree *d*_*min*_	$2d_{min}-n+2\leq \lambda_{2}\leq \frac{n}{n-1}d_{min}$
Maximal degree *d*_*max*_ and diameter Δ	$1-2\frac{\sqrt{\Delta -1}}{\Delta }(\frac{d_{max}-2}{d_{max}})+\frac{2}{d_{max}}\leq \lambda_{2}$
Edge connectivity *e*(*G*)	$2e\left(G\right)(1-\text{cos}\frac{\pi }{n})\leq \lambda_{2}$

*Except for a complete graph $K_{n}$.

## Problem description

We consider a multi-agent system consisting of *n* agents whose communication network topology can be described by a time-varying weighted undirected graph. An agent can represent a simulated or real entity, for example, a sensor in a wireless sensor network or a robot in a multi-robot system. Throughout the paper, we focus on an abstract definition of an agent and use the terms agent, node, and vertex interchangeably. Exact modeling specifications are given in the following assumption.

**Assumption 1**. For a communication network of a multi-agent system, the following holds:

1. At discrete time step *k*, the topology is described by a symmetric adjacency matrix without self-loops, denoted by **A**(*k*).
2. The weights of the adjacency matrix *a*_*ij*_ ∈ [0, 1] represent the quality of the communication channel between agents *i* and *j*.
3. The system connectivity is measured by the second smallest eigenvalue of the Laplacian matrix of the graph—the algebraic connectivity λ_2_.
4. The reference connectivity is given by an external controller, which sets the optimal value depending on the application.

Higher connectivity values are required when there is an increased demand for system performance, such as rapidly disseminating important new information over the network or performing complex distributed computations. On the other hand, when the performance requirements are low, increased connectivity can negatively affect the system due to communication interference and increased energy consumption. Therefore, the goal of topology control and connectivity tracking is to dynamically adjust the agents’ communication network and keep its λ_2_ at the desired value.

In dynamic distributed systems, where agents can only exchange information with their neighbors and links are affected by failures, noise, and obstacles, following the given connectivity reference is a nontrivial problem. In the absence of global topology information, each agent *l* must compute its local estimate of the graph’s adjacency matrix **A**^*l*^(*k*) in the discrete communication interval *k*. By communicating with neighbors, the missing information can propagate through the network and eventually converge to the true joint adjacency matrix **A**,

$$\begin{array}{c} {\text{A}}^{l}\left(k\right)=\text{A},\forall l,k\to \infty . \end{array}$$ (3)

Designing a protocol to ensure fast convergence of (3) under dynamic link changes is the first problem we address in this paper.

With a good estimate of their underlying communication network, the agents can calculate the current λ_2_ and undertake the necessary actions to maintain connectivity—establish new links to increase it or remove the existing ones to decrease it. Which links should be added or deleted, when, and how to ensure the graph remains connected at all times is the second problem we discuss herein.

## Topology estimation

Each agent in the system starts with a partial view of the overall communication graph consisting only of its immediate neighbors. To obtain true and complete knowledge of the entire network, the information about the remaining active links must arrive from others and be incorporated in the local adjacency matrix estimate. The simplest rule for updating the elements of **A**^*l*^(*k*) is in the form of

$$\begin{array}{c} a_{ij}^{l}\left(k+1\right)=a_{ij}^{l}\left(k\right)+\Delta a_{ij}^{l}\left(k\right), \end{array}$$ (4)

where $a_{ij}^{l}\in \left[0,1\right]$ is the quality of the communication link between the agent *i* and *j*, perceived by agent *l* (hence, $a_{ij}^{l}$ is an element of adjacency matrix **A**^*l*^).

To ensure accurate estimation of the adjacency matrix in a dynamic system, the update term $\Delta a_{ij}^{l}\left(k\right)$ must reflect the missing information from the neighbors as well as continuous channel quality measurements. This way, **A**^*l*^ becomes a function of both the environment perceived by the agent *l* and the beliefs of other, non-neighboring agents in the group.

### Communication quality measurements

At each iteration *k* of the consensus algorithm for topology estimation, the agents exchange the local estimates of the global adjacency matrix with their neighbors. The measured quality of the communication channel between agents *i* and *j*, as observed by the agent *l*, is defined as a ratio of successfully received messages over a rolling window of size N*,

$$\begin{array}{c} \tau_{ij}^{l}\left(k\right)=\frac{1}{N^{*}}\sum_{q=k-N^{*}+1}^{k}r_{ij}^{l}\left(q\right), \end{array}$$ (5)

where $r_{ij}^{l}\left(q\right)\in \left\{0,1\right\}$ indicates whether agent *j* received the message from agent *i* in step *q*, as observed by agent *l*.

This model is easy to implement and considers the real-world challenges of message transmission, including channel noise, dropouts, multi-pathing, and delays that exceed the specified communication period. To reduce sensitivity to the number of dropped messages, the window size N* can be increased, and *vice versa*. Additionally, the model can be expanded to handle multiple messages per communication period without difficulty. The consensus protocol for updating the local estimate of the adjacency matrix described in the next section does not depend on the actual measurement model as long as $\tau_{ij}^{l}\in \left[0,1\right]$ so other models can be easily used as well.

When it comes to allowing agent *l* to access the measurements between agents *i* and *j*, it’s important to note that in a realistic scenario, only the agents involved in the data exchange can measure the communication quality. Additionally, an agent can only measure the success rate of incoming messages and must assume that the channel is symmetric. As a result, it is typically the case that *l* = *i* (or *l* = *j*) and

$$\begin{array}{c} \tau_{ij}^{i}=\tau_{ji}^{i}\neq \tau_{ij}^{j}=\tau_{ji}^{j}. \end{array}$$ (6)

### Adjacency matrix updates

Consensus protocol is a commonly used tool for reaching an agreement in distributed systems without global knowledge. General discrete consensus protocol takes the form of

$$\begin{array}{c} \text{x}(k+1)=\text{Gx}(k) \end{array}$$ (7)

or in the iterative version:

$$\begin{array}{c} x_{i}\left(k+1\right)=g_{ii}\cdot x_{i}\left(k\right)+\sum_{j\in N_{i}}g_{ij}x_{j}\left(k\right). \end{array}$$ (8)

The state vector **x**(*k*) represents the current state of the system, e.g., a robot’s position or the ambient temperature measured by the agent. The matrix **G** is a control law matrix and it should be designed so that the system achieves the desired behavior. For the average consensus, which we use to gather communication link qualities that cannot be directly measured, **G** must be designed such that for any initial value **x**(0), **x**(*k*) converges to the average vector $\bar{\text{x}}=(\left(1/n\right)11^{T})\text{x}\left(0\right)$ [56]. In other words, **G** should follow

$$\begin{array}{c} \underset{k\to \infty }{\text{lim}}{\text{G}}^{k}=\left(1/n\right)11^{T}. \end{array}$$ (9)

A good choice for **G** that satisfies (9) is **G** = **I** − *σ* **L** [56]. Since **L** = **D** − **A**, it can be shown that substituting **G** in (8) results in

$$\begin{array}{c} x_{i}\left(k+1\right)=x_{i}\left(k\right)+\sigma \sum_{j\in N_{i}}a_{ij}\left(k\right)\left[x_{j}\left(k\right)-x_{i}\left(k\right)\right], \end{array}$$ (10)

where *σ* is an update step size, and *a*_*ij*_, the element of the adjacency matrix, represents the level of relation between two agents, e.g., trust, attraction, or in our case, communication quality. To capture the dynamic changes in link qualities, general control law (10) needs to be extended with an additional term to update the current estimate based on quality measurements:

$$\begin{array}{c} \epsilon_{ij}^{l}\left(k\right)=\tau_{ij}^{l}\left(k\right)-a_{ij}^{l}\left(k\right). \end{array}$$ (11)

Referring to (6) and (11), if *i* and *j* do not communicate (*l* ≠ *i* ≠ *j*), the measurements of the transmitted and received data packets are not available and we set $\epsilon_{ij}^{l}\left(k\right)=0\;\forall l$.

Note that our goal is to estimate the topology of a multi-agent system. Therefore, we assign the quality of the communication link to both the relationship property and the state in the consensus protocol, since it simultaneously represents our interest and affects the consensus performance. This is in line with the research on a trust-based consensus algorithm presented in [21]. The authors assigned a trust value to each link in the network and introduced an algorithm that ensures the convergence of both the agents’ states and trusts to a common value. From a mathematical point of view, the communication link quality can be treated similarly to trust in that algorithm.

Using the elements of the adjacency matrix as state variables and combining the average consensus protocol (10) with additional measurements (11) we derive the following control law:

$$\begin{array}{c} a_{ij}^{l}\left(k+1\right)=a_{ij}^{l}\left(k\right)+\sigma_{l}\left(k\right)[\sum_{p\in N_{l}}a_{lp}^{l}\left(k\right)[a_{ij}^{p}\left(k\right)-a_{ij}^{l}\left(k\right)]+\epsilon_{ij}^{l}\left(k\right)],\;i\neq j, \end{array}$$ (12)

where $a_{lp}^{l}$ is the agent *l*’s own view on the quality of the communication between it and its neighbor, and $a_{ij}^{p}$ and $a_{ij}^{l}$ are the current estimates of the edge weight between agents *i* and *j*, by agents *p* and *l* respectively. In general, we allow each agent to have its independent time-varying value of *σ*_*l*_(*k*). Assuming the initial underlying communication graph contains a spanning tree, the update equation guarantees that (3) holds. For a detailed derivation of the control law and proof of convergence, we refer the reader to [50].

With the up-to-date estimate of the adjacency matrix **A**^*l*^(*k*), the agents can calculate the corresponding Laplacian matrix and the current algebraic connectivity $\lambda_{2}^{l}\left(k\right)$.

### Update step adaptation

From the control law (12), it is clear that the parameter *σ* has an important influence on the convergence of the system. If *σ* is very small, the future estimate of the edge weight *a*_*ij*_(*k* + 1) will mainly depend on the previous value, and convergence will be slow. On the other hand, making *σ* too large may lead to erratic changes and divergence. The optimal value of *σ* depends heavily on the number of nodes in the graph and how they are connected. Therefore, to ensure the stable behavior of the algorithm in all situations, the initial *σ* must be small which would limit the performance. We propose an adaptation mechanism that monitors the current network of the multi-agent system and adjusts the value of *σ* to keep it at the optimal level.

To determine the optimal value of *σ* (here we omit the agent index *l* and step iteration *k* for brevity), we start by writing the proposed control law in the standard form using *x*_*i*_ as the state variable of agent *i*. Measurement *τ*_*i*_ is considered as an input to the system (*u*_*i*_):

$$\begin{array}{c} x_{i}\left(k+1\right)=x_{i}\left(k\right)+\sigma [\sum_{j\in N_{i}}a_{ij}(x_{j}\left(k\right)-x_{i}\left(k\right))+\underset{\varepsilon_{i}\left(k\right)}{\underset{︸}{\left(u_{i}\left(k\right)-x_{i}\left(k\right)\right)}}]. \end{array}$$ (13)

In matrix form, the update rule can be written as

$$\begin{array}{c} \begin{array}{cc} \text{x}(k+1) & =(\text{I}-\sigma \text{L})\text{x}\left(k\right)+\sigma (\text{u}(k)-\text{x}(k))\\ & =[\text{I}-\sigma (\text{I}+\text{L})]\text{x}\left(k\right)+\sigma \text{u}\left(k\right). \end{array} \end{array}$$ (14)

The transfer function of the system with difference Eq (14) is

$$\begin{array}{c} \frac{X(z)}{U(z)}=\frac{\frac{\sigma }{z-1}}{1+\frac{\sigma }{z-1}\left(\text{I}+\text{L}\right)}=\frac{\sigma }{z-1+\sigma (\text{I}+\text{L})}. \end{array}$$ (15)

The stability of discrete-time LTI (linear time-invariant) systems such as (15) depends on the locations of system poles which are the roots of the characteristic equation, in this case,

$$\begin{array}{c} 0=z\text{I}-\text{I}+\sigma (\text{I}+\text{L}). \end{array}$$ (16)

Since the Laplacian matrix is real and symmetric, it can be diagonalized, and the system can be separated into independent subsystems according to the eigenvalues of **L**. The characteristic equation corresponding to the eigenvalue λ_*i*_ is then

$$\begin{array}{c} 0=1+\left(\lambda_{i}+1\right)\frac{\sigma }{z-1}. \end{array}$$ (17)

In addition to stability, the maximum magnitude of the system poles determines the convergence rate of the consensus:

$$\begin{array}{c} \rho =\underset{p=1,\ldots ,n}{\text{max}}\left|z_{p}\right|. \end{array}$$ (18)

Using the root locus technique common in the analysis of LTI systems applied to (17), we can observe that the pole starts at *z* = 1 and moves left as λ = λ_*i*_, *i* = 1, …, *n* increases. The minimum *ρ* (fastest convergence rate) is achieved when the poles related to the smallest and the largest eigenvalue are equally distant from the center of the unit circle, i.e., when

$$\begin{array}{c} \begin{array}{cc} \lambda_{i}=\lambda_{1}=0 & \Rightarrow z=\rho \\ \lambda_{i}=\lambda_{n} & \Rightarrow z=-\rho . \end{array} \end{array}$$ (19)

Substituting λ_*i*_ and *z* in (17) with conditions in (19) and solving for *σ* and *ρ*, we get the optimal convergence rate ρ* and optimal σ* for which that convergence rate is achieved

$$\begin{array}{cc} \rho^{*}=\frac{\lambda_{n}}{\lambda_{n}+2}, & \;\sigma^{*}=\frac{2}{\lambda_{n}+2}. \end{array}$$ (20)

Using the same set of equations and a condition that *ρ* = max_*p*=1,…,*n*_ |*z*_*p*_| < 1, we can additionally determine the allowed range of values for *σ* that ensure the stability of the consensus:

$$\begin{array}{c} 0<\sigma <\frac{2}{\lambda_{n}+1}. \end{array}$$ (21)

**Fig 3:**
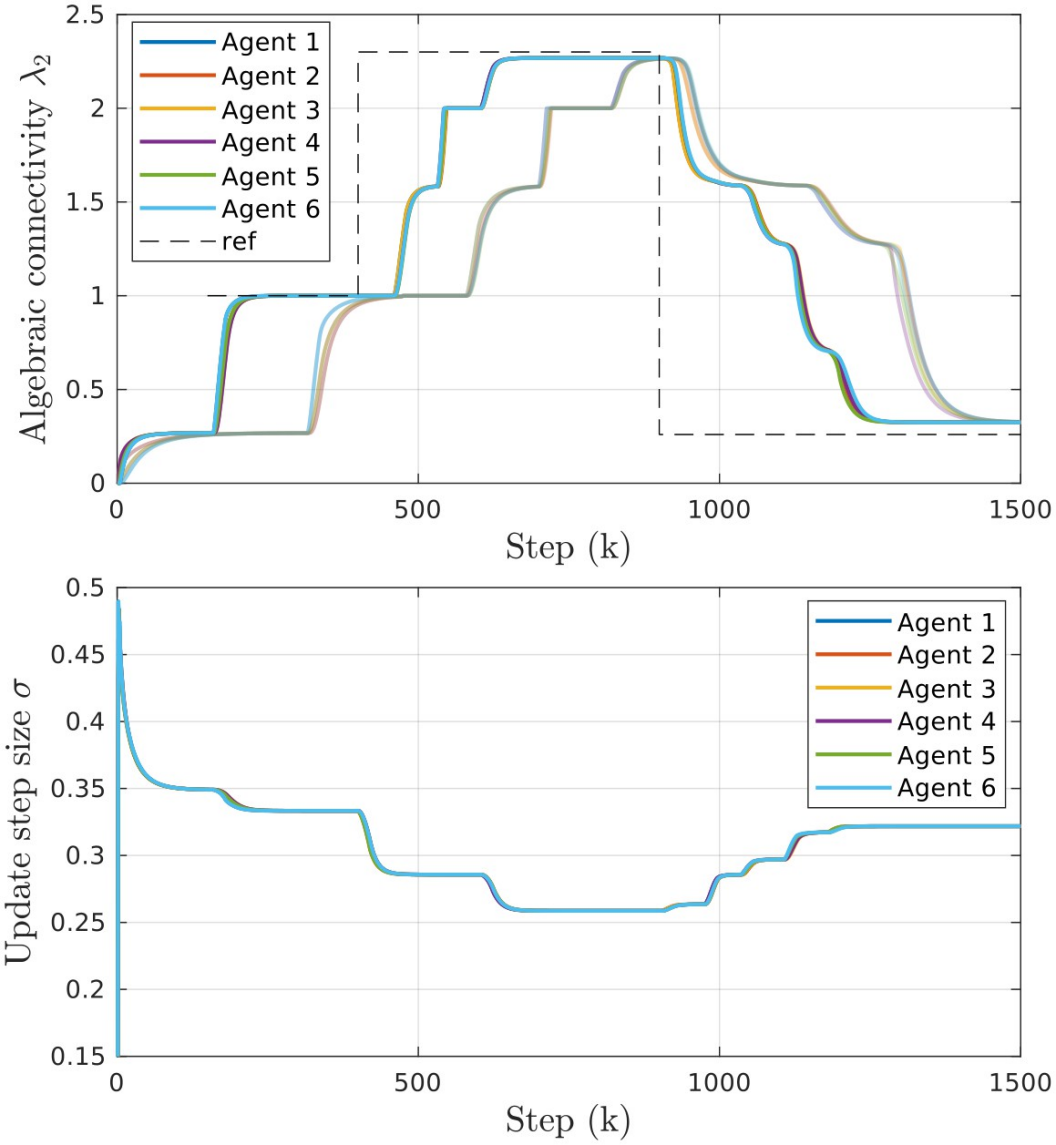
Effectiveness of the proposed *σ* adaptation method. *Top*: Comparison of the effects of different update step sizes in a group of 6 agents. Lighter colors correspond to a fixed *σ* = 0.15, while darker colors represent the case with adaptive *σ*. *Bottom*: Value of update step size *σ* with enabled adaptation.

We can calculate the value of $\lambda_{n}^{l}\left(k\right)$ together with $\lambda_{2}^{l}\left(k\right)$ in every step, which allows us to continuously update the optimal update step $\sigma_{l}^{*}\left(k\right)$. As a result, we are able to achieve fast convergence independent of the topology size and structure. Fig 3 illustrates the effect of the adaptation procedure by showing the estimated algebraic connectivity of each agent over time. In the beginning, the agents are only aware of their immediate neighbors, which do not form a connected graph, and the estimated connectivity is zero. Until *k* = 150, they continue to estimate the initial communication graph defined as a line. Afterward, the connectivity controller is enabled, and the agents collectively add and remove links to track the reference value of connectivity. Lines with stronger colors indicate that *σ* adaptation is enabled, and demonstrate much faster performance compared to the case when the update step is set to the fixed value of *σ* = 0.15.

The proposed complete algorithm for adjacency matrix estimation is summarized in Algorithm 1 and Algorithm 2.

**Algorithm 1**: The pseudo-code for calculating the algebraic connectivity and optimal update step value.

**Input** : **A**^*l*^(*k*)

**Output**: $\lambda_{2}^{l}\left(k\right)$, $\lambda_{n}^{l}\left(k\right)$, $\sigma_{l}^{*}\left(k\right)$

${\text{D}}^{l}\left(k\right)=\text{diag}(\sum_{j=1}^{n}a_{ij}^{l}\left(k\right))$

**L**^*l*^(*k*) = **D**^*l*^(*k*) − **A**^*l*^(*k*)

$\lambda_{2}^{l}\left(k\right),\;\lambda_{n}^{l}\left(k\right)=\text{eig}\left({\text{L}}^{l}\left(k\right)\right)$

$\sigma_{l}^{*}\left(k\right)=2/(\lambda_{n}^{l}\left(k\right)+2)$

**Algorithm 2**: The pseudo-code of the proposed adjacency matrix estimation on agent *l*.

**Input** : $a_{ij}^{l}(k)\in {\text{>A}}^{l}(k)$

$a_{ij}^{p}(k)\in {\text{A}}^{p}(k),p\in N_{l}$

$r_{ij}^{l}[k-N^{*}+1,k],i,j\in [1,n]$

$\sigma_{l}^{*}(k)$

**Output**: $a_{ij}^{l}\left(k+1\right)\in {\text{A}}^{l}\left(k+1\right)$

**foreach** *i* ∈ [1, *n*] **do**

 **foreach** *j* ∈ [1, *n*] **do**

  Calculate quality measurements:

$\tau_{ij}^{l}\left(k\right)=\frac{1}{N^{*}}\sum_{q=k-N^{*}+1}^{k}r_{ij}^{l}\left(q\right)$

  **if** *l* ≠ *i* **or** *l* ≠ *j* **then**

$\epsilon_{ij}^{l}\left(k\right)=0$

  **else**

$\epsilon_{ij}^{l}\left(k\right)=\tau_{ij}^{l}\left(k\right)-a_{ij}^{l}\left(k\right)$

  **end**

  Calculate the update term:

$\Delta a_{ij}^{l}\left(k\right)=\sum_{p\in N_{l}}a_{lp}^{l}\left(k\right)\left(a_{ij}^{p}\left(k\right)-a_{ij}^{l}\left(k\right)\right)+\epsilon_{ij}^{l}\left(k\right)$

  Calculate the new estimates of adjacency matrix elements:

$a_{ij}^{l}\left(k+1\right)=a_{ij}^{l}\left(k\right)+\sigma_{l}^{*}\left(k\right)\cdot \Delta a_{ij}^{l}\left(k\right)$

 **end**

**end**

## Connectivity control

The purpose of the connectivity controller is to enable a multi-agent system to reconfigure its communication topology and dynamically track the desired connectivity. An external module provides the reference value, which depends on the need for enhanced performance or lower energy consumption. In each step, the controller takes the difference between the desired connectivity and the estimated value of algebraic connectivity as input:

$$\begin{array}{c} e_{\lambda_{2}}^{l}\left(k\right)=\lambda_{2,ref}-\lambda_{2}^{l}\left(k\right). \end{array}$$ (22)

As discussed earlier, the relationship between algebraic connectivity and the number, weight, and position of the edges is complex. Consequently, the set of all possible values in a graph with *n* nodes is discontinuous [53], and implementing linear controllers based on formal design methods is therefore not feasible. Instead, we propose a simple symmetric three-level relay controller with parameter $K_{\lambda_{2}}$ that ensures satisfactory tracking and avoids cyclic addition and removal of edges, i.e.,

$$\begin{array}{c} |e_{\lambda_{2}}^{l}\left(k\right)|<K_{\lambda_{2}},\;k\to \infty . \end{array}$$ (23)

If the tracking error is larger than the specified $K_{\lambda_{2}}$, a link should be removed to decrease $\lambda_{2}^{l}\left(k\right)$ toward the reference value. Similarly, if $e_{\lambda_{2}}^{l}<-K_{\lambda_{2}}$, a new link must be added to the graph.

### Adding and removing communication links

After deciding to add or remove a link, each agent should try to select the optimal link to minimize the time required to adopt the required topology and the energy spent on establishing new connections. To determine the effect the modified link will have on the network, we use a heuristic rule based on the elements of the Fiedler vector. The control period *κ* is set to a larger value than the convergence time of the consensus. This allows the agents to have the same state and can deterministically select the link in a decentralized manner. The relation between the Fiedler vector and algebraic connectivity change under perturbation (such as adding a link) is given in the following lemma.

**Lemma 1**. *The first-order approximation of the algebraic connectivity change under perturbation is*:

$$\begin{array}{c} \frac{\partial \lambda_{2}}{\partial l_{ij}}={\text{f}}^{T}\frac{\partial \text{L}}{\partial l_{ij}}\text{f}={\left(f_{i}-f_{j}\right)}^{2}, \end{array}$$ (24)

*where **f** is a normalized eigenvector of the Laplacian, also known as Fiedler vector, such that* **Lf** = λ_2_**f**, *and l*_*ij*_ *is a changing element of the Laplacian matrix*.

*Proof*. A small change in edge weight *a*_*ij*_ will cause a change in *l*_*ij*_. We can denote the corresponding change in the Laplacian matrix as **L**′ = ∂**L**/∂*l*_*ij*_. From the perturbation theory of symmetric matrices [57, Theorem 2.3], we have

$$\begin{array}{c} \frac{\partial \lambda_{2}}{\partial l_{ij}}=\frac{{\text{f}}^{\text{T}}{\text{L}}^{\prime }\text{f}}{{\text{f}}^{T}\text{f}}. \end{array}$$ (25)

Since the Fiedler vector is normalized, **f**^*T*^**f** = 1. From [54, Lemma 13.1.5], we have that

$$\begin{array}{c} {\text{f}}^{\text{T}}{\text{L}}^{\prime }\text{f}=\sum_{i,j}{\left(f_{i}-f_{j}\right)}^{2}, \end{array}$$ (26)

Combined, we get that the change in algebraic connectivity due to the change in the link *a*_*ij*_ is proportional to the difference of the corresponding elements of the Fiedler vector:

$$\begin{array}{c} \frac{\partial \lambda_{2}}{\partial l_{ij}}={\left(f_{i}-f_{j}\right)}^{2}, \end{array}$$ (27)

We can use this result to introduce the rule for selecting the appropriate links for addition and removal.

**Theorem 2** (Analytic model for adding and removing links). *To maximize the likelihood of achieving the desired configuration in the shortest possible time with minimal oscillations and energy expenditure, links should be added according to*

$$\begin{array}{c} {\left(ij\right)}^{+}\left(\kappa \right)=\underset{i,j:\;e_{ij}\notin E\left(\kappa \right)}{\text{arg}\;\text{max}}{\left(f_{i}\left(\kappa \right)-f_{j}\left(\kappa \right)\right)}^{2}, \end{array}$$ (28)

*and removed according to*

$$\begin{array}{c} {\left(ij\right)}^{-}\left(\kappa \right)=\underset{i,j:\;e_{ij}\in E\left(\kappa \right)}{\text{arg}\;\text{min}}{\left(f_{i}\left(\kappa \right)-f_{j}\left(\kappa \right)\right)}^{2},\;\left(f_{i}\left(\kappa \right)-f_{j}\left(\kappa \right)\right)\neq 0. \end{array}$$ (29)

*Proof*. To minimize the number of links and the time required to reach reference connectivity, each added link should cause the largest possible increase in λ_2_. From Theorem 1 we know that (24) approximates the change in λ_2_ caused by changes in *l*_*ij*_ (*a*_*ij*_). Thus, by choosing the link for which (*f*_*i*_ − *f*_*j*_)^2^ has the largest value, we maximize the likelihood that connectivity increases as much as possible. It should be noted that adding more terms of the Taylor series expansion of (24) could sometimes improve the approximation and lead to better connectivity, but it would also significantly increase the computational complexity.

When removing a link, we look for the minimum non-zero change in (*f*_*i*_ − *f*_*j*_)^2^ to avoid the possibility of accidentally disconnecting the graph and to minimize oscillations around the reference value.

To further minimize the likelihood of accidentally disconnecting the link that would reduce connectivity too much or even disconnect the graph, the controller additionally checks the minimum condition for desired connectivity.

**Corollary 2.1**. *To achieve the topology with desired algebraic connectivity* λ_2,*ref*_, *the minimum degree of the graph should be*

$$\begin{array}{c} d_{min}^{*}\geq \frac{n-1}{n}\lambda_{2,ref}. \end{array}$$ (30)

*Proof*. The bound for the minimum degree of the graph directly follows from the lower and upper bounds of the algebraic connectivity defined in Table 2:

$$\begin{array}{c} 2d_{min}-n+2\leq \lambda_{2}\leq \frac{n}{n-1}d_{min}. \end{array}$$ (31)

Finally, we return to the definition of the controller parameter $K_{\lambda_{2}}$.

**Theorem 3** (Lower bound of $K_{\lambda_{2}}$). *For* (23) *to hold, the relay parameter should satisfy*

$$\begin{array}{c} K_{\lambda_{2}}>\delta_{m}\cdot \underset{i,j:\;e_{ij}\in E\left(\kappa \right)}{\text{max}}{\left(f_{i}-f_{j}\right)}^{2} \end{array}$$ (32)

*where δ*_*m*_ ∈ [0, 1] *is a user-defined maximum allowable variation in link quality, and f*_*i*_ *and f*_*j*_ *are the i-th and j-th elements of the normalized Fiedler vector* **f**.

*Proof*. We know from Lemma 1 that for a small change *δ* in *l*_*ij*_ (*a*_*ij*_), the change in λ_2_ is proportional to the difference in the corresponding Fiedler vector components, $\Delta_{\lambda_{2}}=\delta {\left(f_{i}-f_{j}\right)}^{2}$. Thus, by finding the maximum difference between pairs of all entries of **f** corresponding to existing edges, we determine the maximum (worst-case) change in λ_2_ caused by a sudden but small change *δ*_*m*_ in the quality of the most sensitive connection

$$\begin{array}{c} \Delta \lambda_{2,max}=\delta_{m}\cdot \underset{i,j:\;e_{ij}\in E\left(\kappa \right)}{\text{max}}{\left(f_{i}-f_{j}\right)}^{2}. \end{array}$$ (33)

Thus, to avoid oscillations in the algebraic connectivity caused by external disturbances of the connections, i.e., to guarantee that (23) is satisfied, the $K_{\lambda_{2}}$ parameter of the relay controller should be larger than the expected Δλ_2,*max*_. The value of *δ*_*m*_ depends on the user-defined tolerance for variations in the connectivity level. We set it to 20% of the maximum connectivity quality, *δ*_*m*_ = 0.2. If the quality change is greater than this value, new links should be added or removed.

### Energy conservation

Depending on the network structure, some links in the graph may not contribute to the total algebraic connectivity. For example, adding a link to a node whose degree is already larger than the $d_{min}^{*}$ or in the case that λ_2_ has multiple repeating values (λ_2_ = λ_3_ = …) will not immediately increase the connectivity. Such links still consume energy and possibly add noise to the channel.

When the estimated value of λ_2_ reaches the commanded reference value and enters a stationary state, the agents can activate a strategy for removing energy-consuming redundant links. The strategy uses the result from Lemma 1 by finding the link (ij)∈E(κ) for which (*f*_*i*_ − *f*_*j*_) = 0.

### Convergence detection

The connectivity controller works best when all agents in the system have approximately the same estimation of their current communication network. That way, they can reach a common decision without excess communication using a deterministic rule for modifying links. Ensuring that all agents reach a consensus before running the connectivity controller can be achieved by allowing a long enough control period *κ* or by detecting when the agents have reached a consensus.

First, every agent calculates the maximum difference between its own and neighboring adjacency matrices in the form of local Frobenius norms

$$\begin{array}{c} e_{\text{A}}^{l}\left(k\right)=\underset{i\in N_{l}}{\text{max}}\{{\left\|{\text{A}}^{l}\left(k-1\right)-{\text{A}}^{i}\left(k-1\right)\right\|}_{F}\}. \end{array}$$ (34)

If the local norm $e_{\text{A}}^{l}\left(k\right)$ is smaller than the set threshold *e*_*min*_, that means that the agent *l* has converged to a common value with its immediate neighbors and would be ready to start another round of connectivity control. However, as other agents may not yet be ready, the local information needs to be propagated through the network.

Each agent tracks its own and other agents’ readiness in a boolean flag vector **r**^*l*^ ∈ {0, 1}^*n*^ and updates it with an OR consensus running in parallel to the main procedure:

$$\begin{array}{c} e_{\text{A}}^{l}\left(k\right)\leq e_{min}\Rightarrow {\text{r}}^{l}(k^{)}=\underset{i\in N_{l}}{⋁}{\text{r}}^{i}\left(k-1\right)\vee \begin{array}{lllll} {}[0 & \ldots & 1 & \ldots & 0],\\ 1 & \ldots & l & \ldots & n \end{array} \end{array}$$ (35)

where ∨ and ⋁ represent element-wise OR operations between the vectors, and 1 and 0 are true and false values respectively. Agents can communicate their readiness vectors along with adjacency matrices in each iteration. When all values in the readiness vector become true,

$$\begin{array}{c} {\text{r}}^{l}=1_{\left[1\times n\right]}, \end{array}$$ (36)

the agent *l* performs the necessary connectivity control procedure.

For a better overview, the pseudocode of the whole proposed connectivity estimation and control method can be found in Algorithm 3.

**Algorithm 3**: The pseudo-code of the proposed connectivity control procedure running on agent *l*.

Initialize adjacency matrix estimate **A**^*l*^(0) with known local connections.

**while** *True* **do**

 Exchange communication link qualities $a_{ij}^{l}$ with neighbors $N_{l}$.

 Update the adjacency matrix estimate **A**^*l*^ according to Algorithm 2.

 Calculate $\lambda_{2}^{l}$ from **A**^*l*^ using Algorithm 1.

 Calculate $e_{\lambda_{2}}^{l}$ from (22).

 **if** *Condition* (36) *is satisfied* **then**

  **if** $e_{\lambda_{2}}^{l}>K_{\lambda_{2}}$ **then**

   Add a link according to (28).

  **else if** $e_{\lambda_{2}}^{l}<-K_{\lambda_{2}}$ **then**

   Remove a link according to (29).

  **else if** $|e_{\lambda_{2}}^{l}\left(k\right)|<K_{\lambda_{2}}$ **then**

   Remove unnecessary link for energy conservation.

  **end**

 **end**

**end**

## Results and discussion

**Fig 4:**
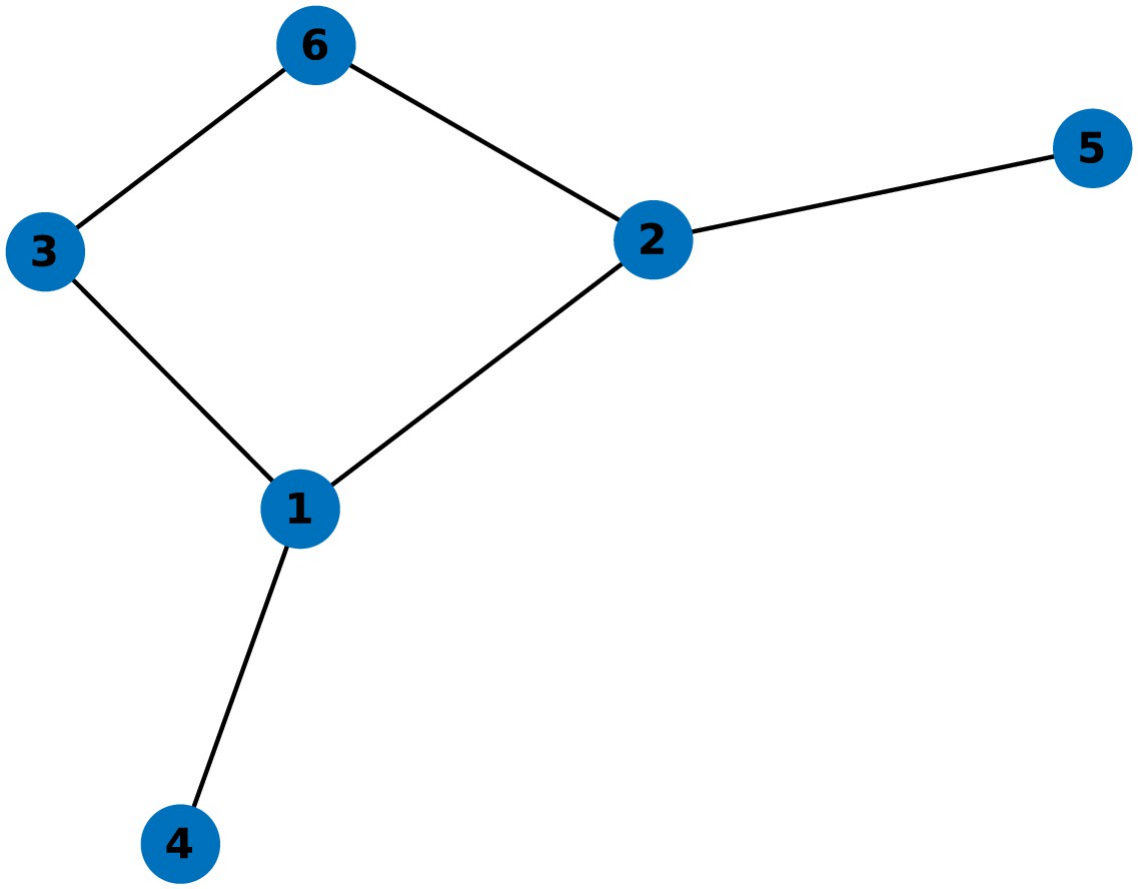
Initial topology of a system with 6 agents.

We demonstrate the effectiveness of the proposed consensus-based method for estimating the adjacency matrix and algebraic connectivity as well as the analytical control law for tracking the desired connectivity profile in extended simulation experiments. The experiments are performed in MATLAB 2023b [58]. We place great emphasis on implementing the algorithm in a truly decentralized manner, where each agent only knows about the connections with its immediate neighbors in the initial communication topology. In the system, information is exchanged between agents solely through simulated communication. We present the experiments conducted with two group sizes, consisting of 6 and 15 agents, whose initial topologies are shown in Figs 4 and 10.

### Adjacency matrix estimation

**Fig 5:**
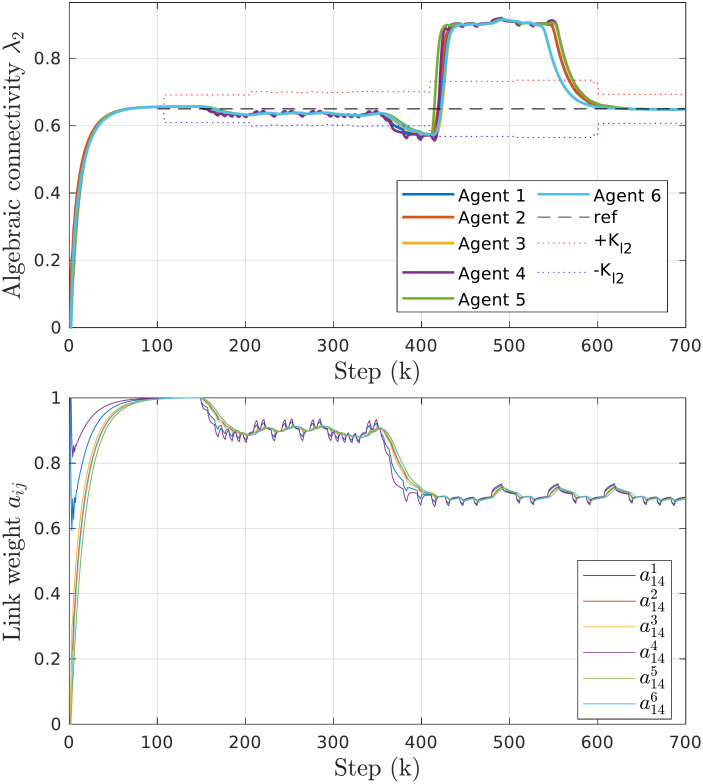
Results of topology estimation experiment. *Top*: Time responses of local algebraic connectivity estimates for the initial topology shown in Fig 4 with bounds of the allowable tracking error. *Bottom*: Local estimates of the link quality between agents 1 and 4.

In the first example, illustrated in Fig 5, we demonstrate the proposed method’s ability to correctly estimate the global network topology and its algebraic connectivity. Each agent starts with a local view of the initial topology shown in Fig 4. As these local connections do not initially form a connected graph, the estimated algebraic connectivity in the first step is zero. Upon exchanging information about their connections, the local adjacency matrices converge quickly to the true value of the global adjacency matrix, thereby satisfying (3).

At step *k* = 150, we simulate a degradation in the communication channel between agents 1 and 4. Specifically, each message has a probability of 90% to arrive successfully at its destination. The affected agents can directly measure the change in the link quality ($\tau_{14}^{1}=\tau_{41}^{4}=0.9$) and are the first to update the estimate of that link to *a*_14_ = *a*_41_ = 0.9 (shown in the bottom part of Fig 5). Other agents converge at different rates depending on their distance from agent 1, as the information propagates through the network using the consensus protocol.

The reduction in quality of the link 1-4 causes a small change in the estimated algebraic connectivity. However, since the change is within the expected *δ*_*m*_ = 0.2 of the link quality, the connectivity tracking error $e_{\lambda_{2}}^{l}$ remains within $K_{\lambda_{2}}$ bounds and no corrective action is necessary. When the quality of the link 1-4 is further reduced to *a*_14_ = *a*_41_ = 0.7 at step *k* = 350, the tracking error exceeds the allowable limit. Consequently, agents decide to reconfigure the network by adding and removing links, resulting in λ_2_ returning to the desired reference value.

### Connectivity tracking

In the following experiments, we demonstrate our method’s ability to adjust the network connectivity based on user-defined reference, recover from unexpected agent failures, and manage redundant links to provide better energy efficiency.

#### Following the connectivity profile

**Fig 6:**
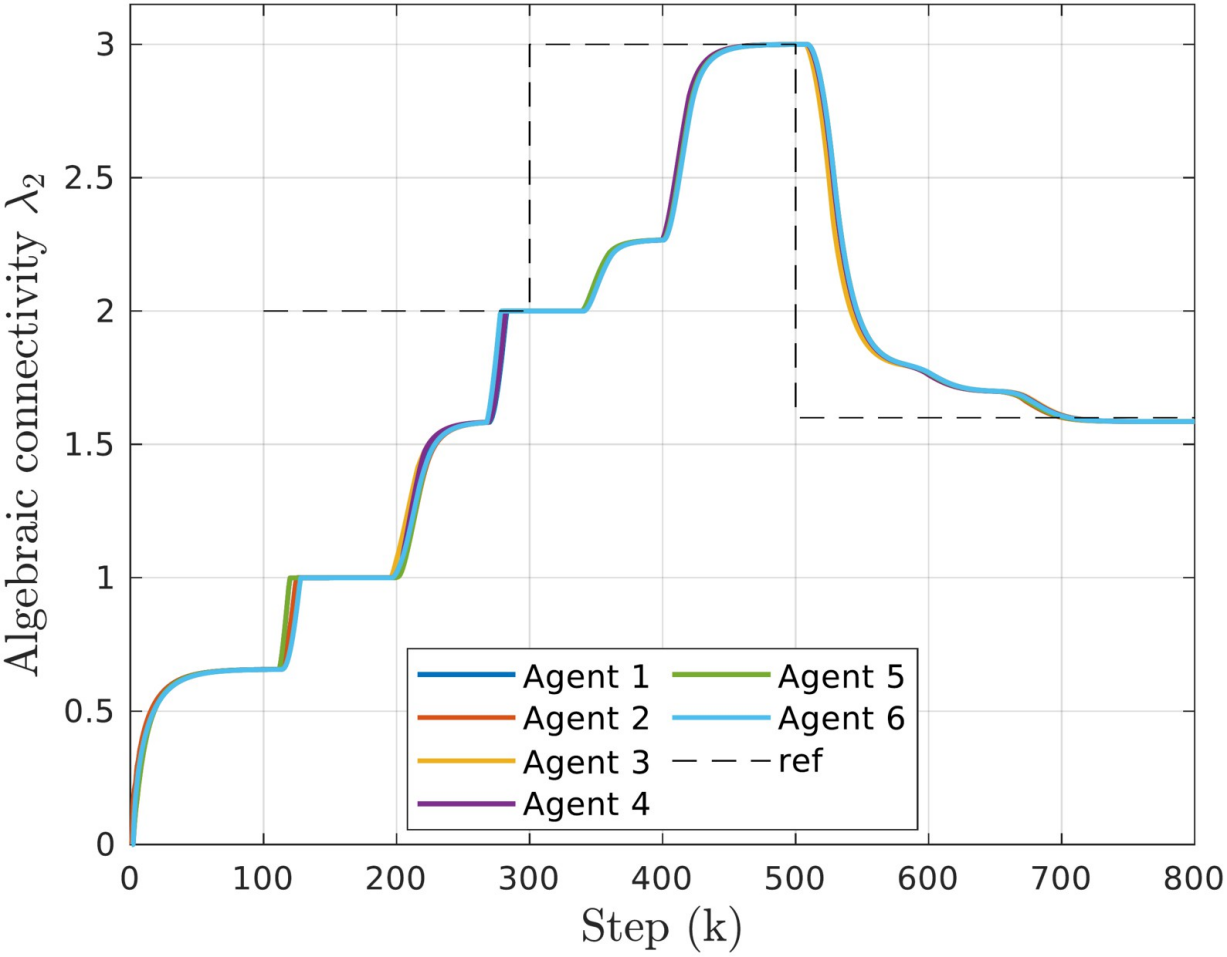
Time responses of algebraic connectivity estimates for the system of 6 agents and changing λ_*ref*_.

**Fig 7:**
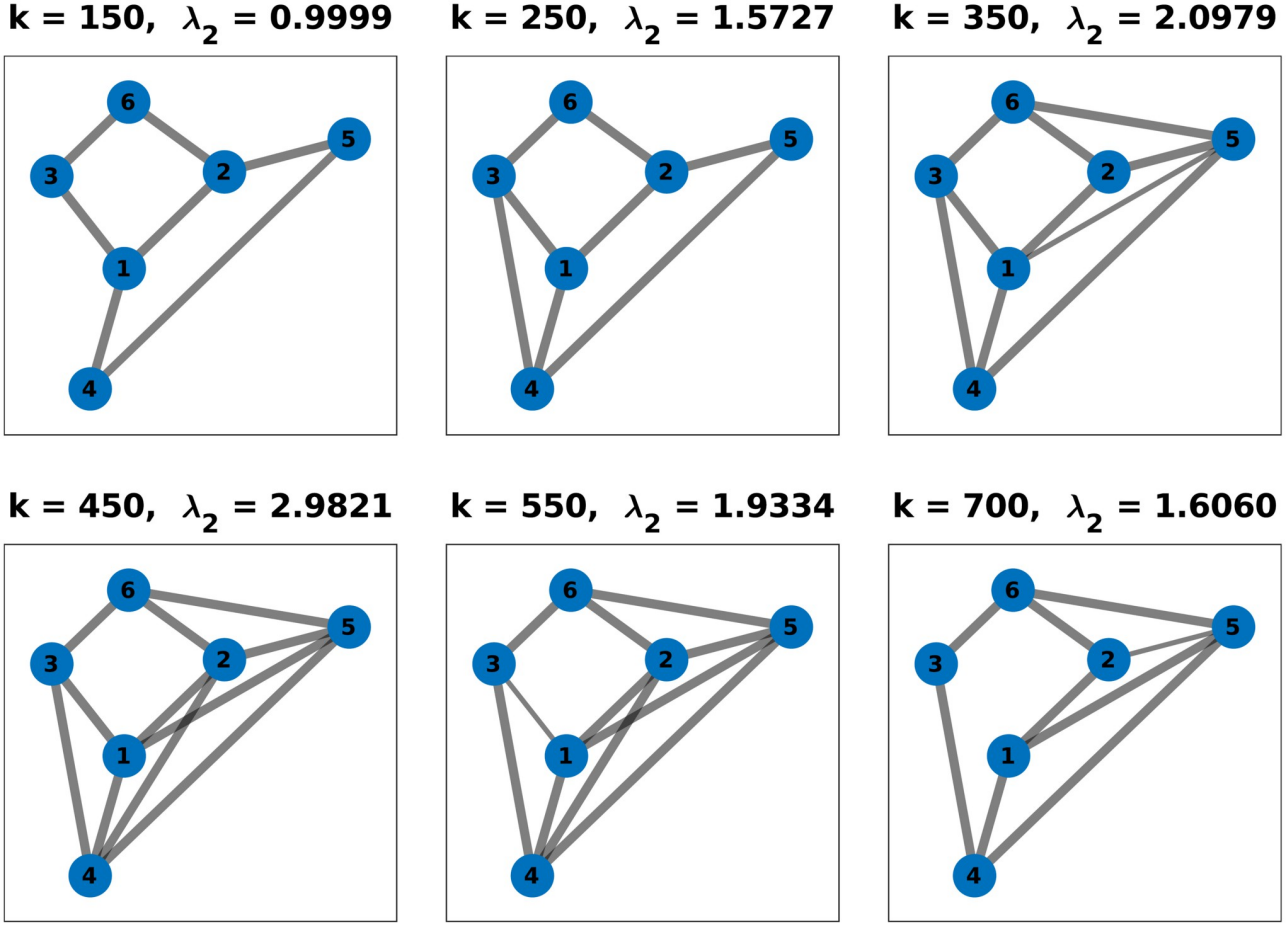
Snapshots of the multi-agent topologies during the experiment with changing algebraic connectivity reference. The thickness of the edges represents the link quality.

Fig 6 shows the estimated values of the algebraic connectivity while tracking the given reference (dashed line). After the first 100 calculation steps, the agents reach a consensus on their initial topology (see Fig 4) and converge to a common value of λ_2_ = 0.6571. At *k* = 100 the reference tracking begins by setting λ_2,*ref*_ = 2. Although several edges need to be added to the graph, the group eventually achieves the commanded connectivity. This process is repeated and the connectivity increases further to λ_2_ = 3. At *k* = 500, the reference value is reduced to 1.6, prompting agents to remove links until the final reference is reached. Snapshots of the network topology at certain steps during the experiment can be found in Fig 7.

#### Resilience to agent failures

**Fig 8:**
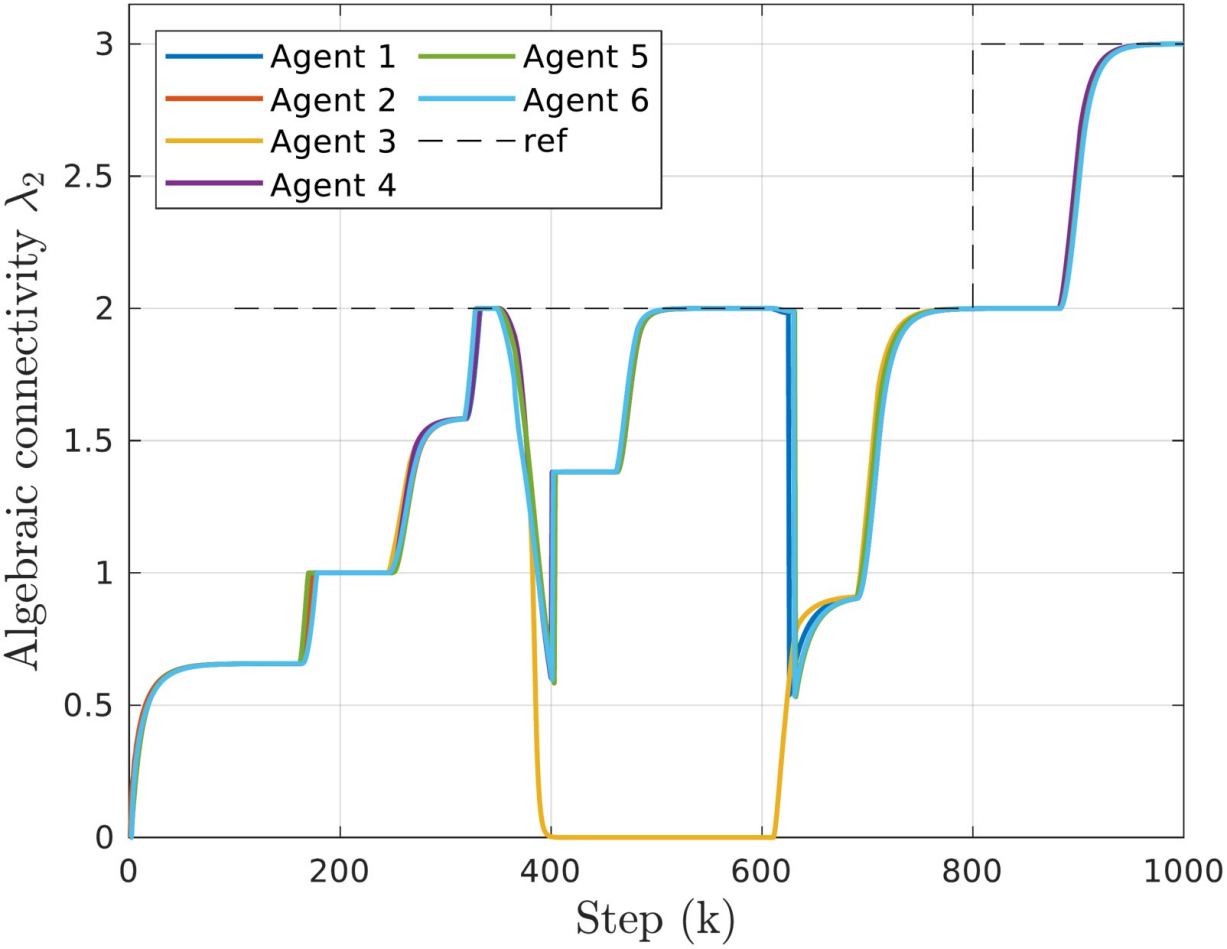
Time responses of algebraic connectivity estimates in case of an agent failure. In the system of 6 agents a failure of agent 3 is simulated at step *k* = 350. The agent rejoins at step *k* = 600.

Depending on the topology structure, agent failures can cause significant changes in connectivity or result in a disconnected communication graph. To prevent this, the proposed method enables the multi-agent system to detect unresponsive neighbors and reconfigure to maintain connectivity. The experiment depicted in Fig 8 starts with a period of initial topology estimation and reaching the reference connectivity λ_2,*ref*_ = 2. At *k* = 350, a simulated failure of agent 3 leads to a sharp drop in algebraic connectivity. The other agents detect this unresponsiveness and exclude agent 3 from future calculations while establishing additional links to maintain connectivity. At *k* = 600, the failed agent becomes responsive again and rejoins the group. Adding a new, poorly connected agent initially reduces the overall λ_2_, but after several rounds of connectivity control, the commanded reference value is restored.

#### Energy conservation

**Fig 9:**
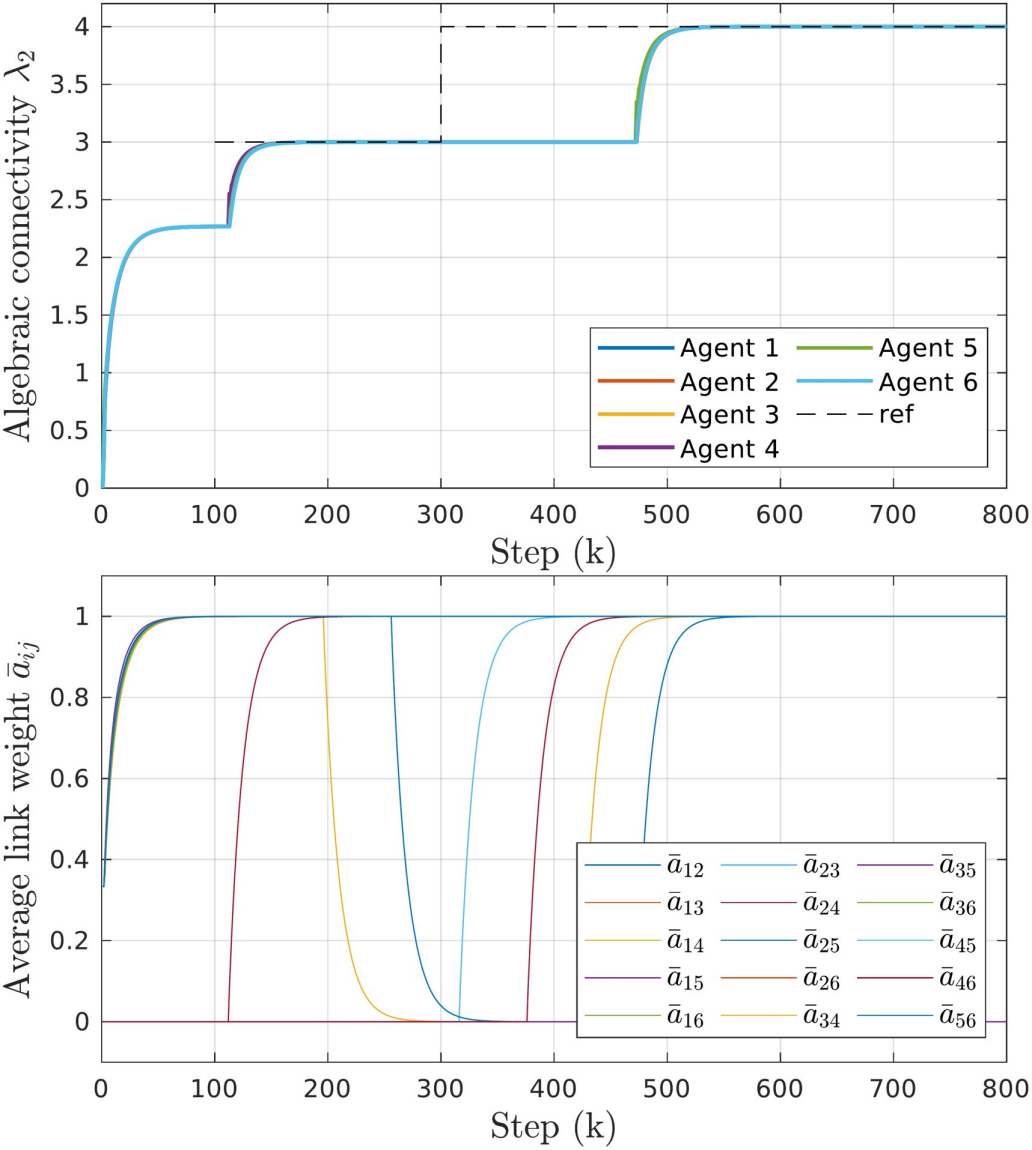
Results of energy conservation experiment. *Top*: Time responses of algebraic connectivity estimates for the system of 6 agents and changing λ_*ref*_. *Bottom*: Average link qualities showing how removing some edges (e.g., 1-4 and 2-5) does not affect the connectivity but reduces energy consumption.

In certain topologies, some links may not contribute to the overall algebraic connectivity but still consume energy. When reference connectivity is reached in steady-state situations, such links can be removed to improve energy efficiency without compromising connectivity. Fig 9 illustrates the time responses of algebraic connectivity for a well-connected group of 6 agents along with the average values of their local estimates of individual link qualities. At *k* = 200, the group reaches the desired connectivity and enters a steady state. The agents collectively determine that links 1-4 and 2-5 are currently unnecessary, leading to their removal. When the reference changes to λ_2,*ref*_(*k* = 300) = 4, the previously removed links must be added before connectivity is further increased.

### Extended scenario

**Fig 10:**
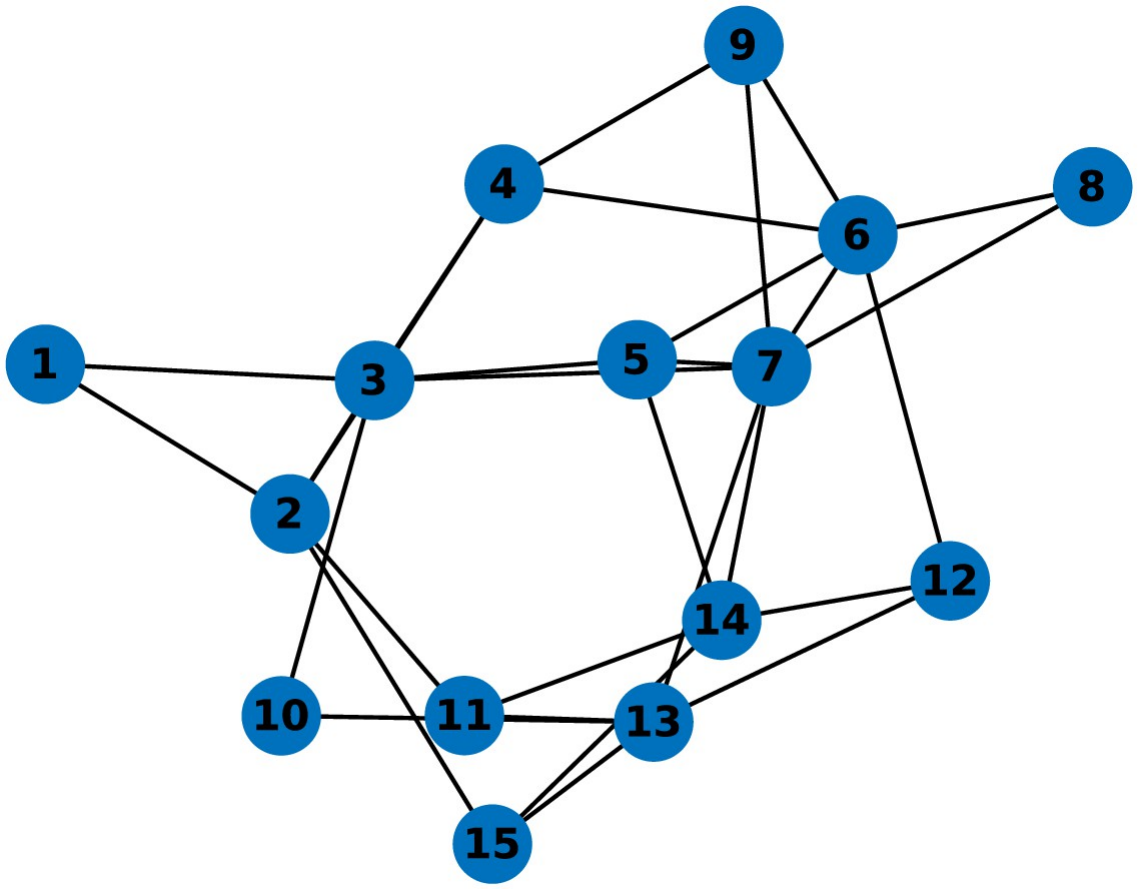
Initial topology of a system with 15 agents.

**Fig 11:**
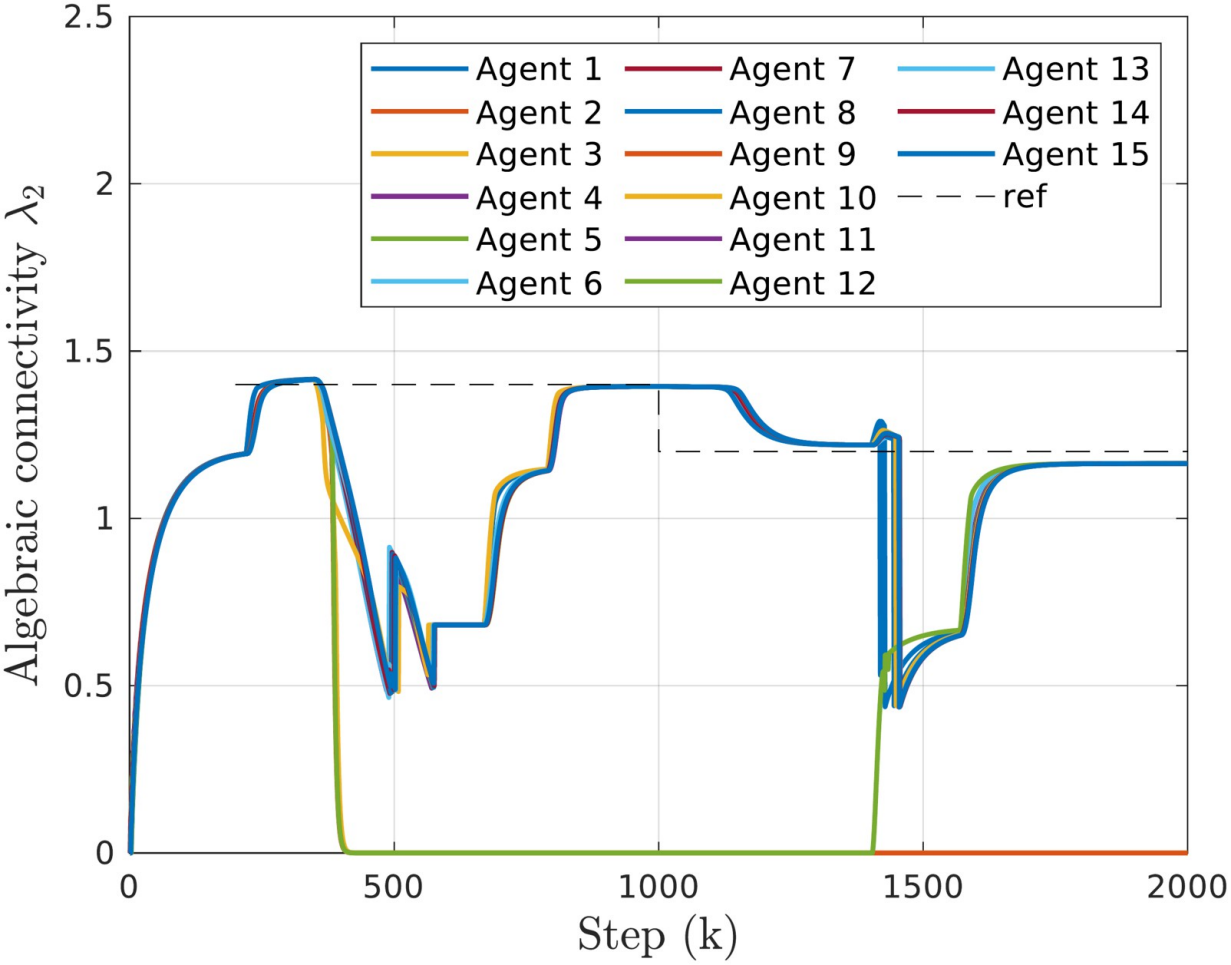
Time responses of algebraic connectivity estimates for the system of 15 agents. A failure of agents 3, 9, and 12 is simulated at step *k* = 350. Agent 12 rejoins at step *k* = 1400.

The extended scenario explores a group of 15 agents, showcasing the effectiveness of the proposed approach for larger systems. The initial topology used in experiments is depicted in Fig 10. We assess the system’s capability to track the reference connectivity profile and its resilience to agent failures. Fig 11 illustrates the time responses of the estimated algebraic connectivity in the given scenario with 15 agents.

Initially, the agents reach a consensus on their starting topology and add a link to increase connectivity to λ_2,*ref*_ = 1.4. At step *k* = 350, a simulated failure of agents 3, 9, and 12 occurs. Similar to the case with a group of 6 agents, this failure results in a sharp decrease in connectivity. Subsequently, depending on the number of neighbors, the failed agents are excluded one by one in rapid succession until the connectivity stabilizes. The remaining agents then reconfigure their topology to restore the commanded connectivity value.

Starting from *k* = 1000, the agents begin following the new reference λ_2,*ref*_ = 1.2. Agent 12 resumes communication in step *k* = 1400 and rejoins the group, causing a change in connectivity. Finally, additional links are created to maintain the desired connectivity.

### Possible extensions and future work

In this study, we addressed the challenge of distributed connectivity tracking in multi-agent systems through reconfiguration of communication links, offering key insights into the self-organization and resilience of such systems. We based our approach on the trust-inspired average consensus protocol, chosen for its simplicity and demonstrated effectiveness—crucial properties for implementation on distributed devices with limited hardware resources.

A potential avenue for future research would be to extend these methods to more complex real-world scenarios, such as networks with limited or quantized communication, as discussed in [50], or communication with long delays. Our current analysis is limited to undirected networks, where bidirectional communication between agents is assumed. A natural extension of this work would involve applying our methods to directed networks and exploring generalized algebraic connectivity, with [27] offering a potential starting point.

The use of the Fiedler vector to select links for modifying the network topology provides an analytical, efficient, and straightforward approach. However, predicting connectivity changes after link modifications remains a challenge, given the non-linear relationship between algebraic connectivity and link weights, which can result in overshooting in the connectivity controller. Our future research will explore alternative link selection strategies, including the use of artificial intelligence and machine learning techniques specifically designed for graph structures, to better predict and manage connectivity dynamics.

## Conclusion

Effective communication is essential in distributed multi-agent systems as it improves performance and collaboration. However, maintaining many connections can be costly and harm overall system efficiency. In scenarios such as coordinating a robotic system’s motion or collecting and processing data in wireless sensor networks, monitoring the current topology and adjusting it to track the desired connectivity can be beneficial. Furthermore, the algorithms used in practical applications must be resilient to agent failures and external disturbances.

In this work, we have presented a distributed method for estimating the communication topology of a multi-agent system and its corresponding algebraic connectivity λ_2_. We used a modified consensus protocol to propagate local information throughout the network and added an adaptation mechanism to speed up the convergence. Additionally, we designed an analytical connectivity controller based on the value of the Fiedler vector to control the connectivity by adding and removing links.

To validate our approach, we have performed several experiments in the simulation with a different number of agents. The experiments show that using only local information, the multi-agent system can accurately estimate individual link qualities and overall graph connectivity. Additionally, the system remains robust against external disturbances and agent failures while adjusting its communication topology to track desired connectivity profiles. Finally, the proposed method can automatically remove links that do not contribute to connectivity but consume energy, making it generally a more complete solution than the existing approaches.
